# Comparative genomic analysis of six new-found integrative conjugative elements (ICEs) in *Vibrio alginolyticus*

**DOI:** 10.1186/s12866-016-0692-9

**Published:** 2016-05-04

**Authors:** Peng Luo, Xiangyan He, Yanhong Wang, Qiuting Liu, Chaoqun Hu

**Affiliations:** Key Laboratory of Tropical Marine Bio-resources and Ecology, South China Sea Institute of Oceanology, Chinese Academy of Sciences, Guangzhou, 510301 China; Guangdong Key Laboratory of Applied Marine Biology, Chinese Academy of Sciences, Guangzhou, 510301 China; South China Sea Bio-Resource Exploitation and Utilization Collaborative Innovation Center, Guangzhou, 510275 China; University of Chinese Academy of Sciences, Beijing, 100049 China

**Keywords:** *Vibrio alginolyticus*, Integrative Conjugative Elements (ICEs), Comparative genomics, Transposable genetic Elements (TEs)

## Abstract

**Background:**

*Vibrio alginolyticus* is ubiquitous in marine and estuarine environments. In 2012–2013, SXT/R391-like integrative conjugative elements (ICEs) in environmental *V. alginolyticus* strains were discovered and found to occur in 8.9 % of 192 *V. alginolyticus* strains, which suggests that *V. alginolyticus* may be a natural pool possessing resourceful ICEs. However, complete ICE sequences originating from this bacterium have not been reported, which represents a significant barrier to characterizing the ICEs of this bacterium and exploring their relationships with other ICEs. In the present study, we acquired six ICE sequences from five *V. alginolyticus* strains and performed a comparative analysis of these ICE genomes.

**Results:**

A sequence analysis showed that there were only 14 variable bases dispersed between ICE*Val*E0601 and ICE*Val*HN492. ICE*Val*E0601 and ICE*Val*HN492 were treated as the same ICE. ICE*Val*A056-1, ICE*Val*E0601 and ICE*Val*HN492 integrate into the 5′ end of the host’s *prfC* gene, and their Int and Xis share at least 97 % identity with their counterparts from SXT. ICE*Val*E0601 or ICE*Val*HN492 contain 50 of 52 conserved core genes in the SXT/R391 ICEs (not *s025 *or *s026*)*.* ICE*Val*A056-2, ICE*Val*HN396 and ICE*Val*HN437 have a different *tRNA-ser* integration site and a distinct int/xis module; however, the remaining backbone genes are highly similar to their counterparts in SXT/R391 ICEs. DNA sequences inserted into hotspot and variable regions of the ICEs are of various sizes. The variable genes of six ICEs encode a large array of functions to bestow various adaptive abilities upon their hosts, and only ICE*Val*A056-1 contains drug-resistant genes. Many variable genes have orthologous and functionally related genes to those found in SXT/R391 ICEs, such as genes coding for a toxin-antitoxin system, a restriction-modification system, helicases and endonucleases. Six ICEs also contain a large number of unique genes or gene clusters that were not found in other ICEs. Six ICEs harbor more abundant transposase genes compared with other parts of their host genomes. A phylogenetic analysis indicated that transposase genes in these ICEs are highly diverse.

**Conclusions:**

ICE*Val*A056-1, ICE*Val*E0601 and ICE*Val*HN492 are typical members of the SXT/R391 family. ICE*Val*A056-2, ICE*Val*HN396 and ICE*Val*HN437 form a new atypical group belonging to the SXT/R391 family. In addition to the many genes found to be present in other ICEs, six ICEs contain a large number of unique genes or gene clusters that were not found in other ICEs. ICEs may serve as a carrier for transposable genetic elements (TEs) and largely facilitate the dissemination of TEs.

**Electronic supplementary material:**

The online version of this article (doi:10.1186/s12866-016-0692-9) contains supplementary material, which is available to authorized users.

## Background

Integrative conjugative elements (ICEs) are self-transmissible mobile genetic elements (MGEs) that play a major role in gene flow in bacterial populations [1]. To date, 460 ICEs have been identified (http://db-mml.sjtu.edu.cn/ICEberg) in a variety of Gram-positive and Gram-negative bacteria [2]. Among them, the SXT/R391 family of ICEs is one of the largest and most studied ICE families and currently consists of 89 members [2, 3]. In 1996, SXT/R391 ICEs were first described in *Vibrio cholerae*, the etiologic agent of the diarrheal disease cholerae, and in *Providencia rettgeri*, which was isolated in 1972 [4, 5]. Since then, SXT/R391 ICEs were widely found to be prevalent in the 7th pandemic isolates of *V. cholerae*, other environmental *Vibrio* species and in some Enterobacteriaceae species [6–10]. A comparative genomic analysis of 13 widely distributed SXT/R391 ICEs indicated that all of these ICEs consist of the same syntenous and nearly identical 52 core genes, whereas other families of closely related mobile elements, such as lambdoid and T4-like phages, exhibit greater diversity [6]. Thus, SXT/R391 ICEs may be a relatively recent creation of evolution [6]. Five hotspots (HS1–HS5) and four variable regions (VR I–IV) are interspaced into the conserved backbone of ICEs, and these variable DNAs generally code for resistance to antibiotics, heavy metals and bacteriophage infection, toxin/antitoxin (TA) systems or c-di-GMP synthesis [6, 7, 11–14], which confer hosts adaptive functions to various environments.

*V. alginolyticus* is ubiquitous in marine and estuarine environments and is the most common *Vibrio* species isolated in southern coastal areas of China [15, 16]. *V. alginolyticus* has received increasing attention because some strains are pathogenic to humans and aquatic animals and have caused huge economic losses [17, 18]. In 2012–2013, SXT/R391-like ICEs in environmental *V. alginolyticus* strains were first discovered [9, 13, 19] and found to occur in 8.9 % of 192 *V. alginolyticus* strains [19]. Due to the ubiquity of *V. alginolyticus* in marine and estuarine environments and because ICEs can transfer at a relatively high frequency between phylogenetically distant species [7, 20–22], it is natural to speculate that *V. alginolyticus* may represent a reservoir for SXT/R391-like ICEs. These results also raise concerns that ICEs will further disseminate and thereby increase the risk of dissemination of multidrug-resistant gene clusters. However, until now no complete ICE sequences in *V. alginolyticus* have been reported. This represents a substantial barrier in characterizing the ICEs of this bacterium to further address the problems mentioned above.

In this study, we acquired six ICE sequences from five *V. alginolyticus* strains including a strain harboring two coresident ICEs to perform a comparative analysis of these ICE genomes. This work will shed light on the characteristics of the ICEs in environmental *Vibrio* species and provides new knowledge of the considerable diversity of genes and potential accessory functions encoded by the variable DNA in these ICEs.

## Results

### Assembly and annotation of the ICEs in *V. alginolyticus*

To obtain the complete sequences of six ICEs, high-throughput HiSeq 2000 sequencing of the genomes of *V. alginolyticus* strains, A056, E0601, HN396, HN437 and HN492 (Table 1), was carried out. Fifty-six, 93, 117, 133 and 103 scaffolds sequences were, respectively, obtained, assembled and annotated using the RAST annotation pipeline [23]. Genome annotation revealed one ICE sequence in each genome of strains E0601, HN396, HN437 and HN492; strain A056 contains two coexistent ICEs. The alignment and assembly of these ICE-related scaffolds were manually performed using the sequences of SXT (the representative ICE of the SXT/R391 family), ICE*Vch*Ind4 and ICE*Vch*Mex1 as references. Gap filling was carried out through PCR followed by sequencing, which yielded the complete sequences of ICE*Val*A056-1, IC*EVal*A056-2, ICE*Val*E0601, ICE*Val*HN396, ICE*Val*HN437 and ICE*Val*HN492. All ICE sequences were deposited in GenBank under the accession numbers KR231688-KR231689 and KT072768-KT072771.

**Table 1:** Features of the ICEs in *Vibrio alginolyticus* strains

ICE	Host strain	Sources	Sites and year of isolation	Size (bp)	Identity to Int_SXT_ (%)	Integration site	Resistant genes	Notable variable genes code for^b^	GenBank accession number
ICE*Val*A056-1	A056	*Litopeneaus vannamei*	Zhanjiang, Guangdong, China, 2003	89004	97	*prfC*	*strBA*, *sul2*	type III RM system, diguanylate cyclase, acriflavin resistance protein, membrane-fusion protein	KR231688
ICE*Val*A056-2				103826	*N* ^a^	*tRNA-ser*	*N*	type I RM system, Fic family protein, HigA, MosTA, **choline uptake protein**, **mechanosensitive channel regulation**, **calcium/sodium proton antiporter**, **oxaloacetate and citrate metabolism**, **PrrABCD**	KR231689
ICE*Val*E0601	E0601	Seawater	Yangjiang, Guangdong, China, 2006	106165	99	*prfC*	*N*	HipBA, **Flp pilus assembly system**, **P pilus assembly system,** type III RM system, **threonine efflux protein**, **choline uptake protein**, diguanylate cyclase, DDE endonuclease, **mechanosensitive channel regulation, RNA-dependent DNA polymerases**	KT072768
ICE*Val*HN492	HN492	Seawater	Sanyan, Hainan, China, 2008	106164	99	*prfC*	*N*		KT072769
ICE*Val*HN396	HN396	Seawater	Qinzhou, Guangxi, China, 2008	86687	*N*	*tRNA-ser*	*N*	type II RM system, Fic family protein, HigA, diguanylate cyclase, **aerotaxis sensor**, **chemotaxis sensor**, DNA recombination-mediator, DDE endonuclease	KT072770
ICE*Val*HN437	HN437	Seawater	Haikou, Hainan, China, 2008	94290	*N*	*tRNA-ser*	*N*	type I RM system, Fic family protein, HigA, **organic hydroperoxide resistance**, DDE endonuclease, **nucleotide metabolism**, **phage lysin**, **rhamnose metabolism**, **sulfate assimilation**, **synthesis of capsular polysaccharide**	KT072771

^a^ *N* represents that compared genes lack similarity or that no resistant genes were found

^b^The functions conferred by unique gene contents that are not found in other ICEs are indicated in boldface

### General genetic structures of the ICEs in *V. alginolyticus*

**Fig. 1:**
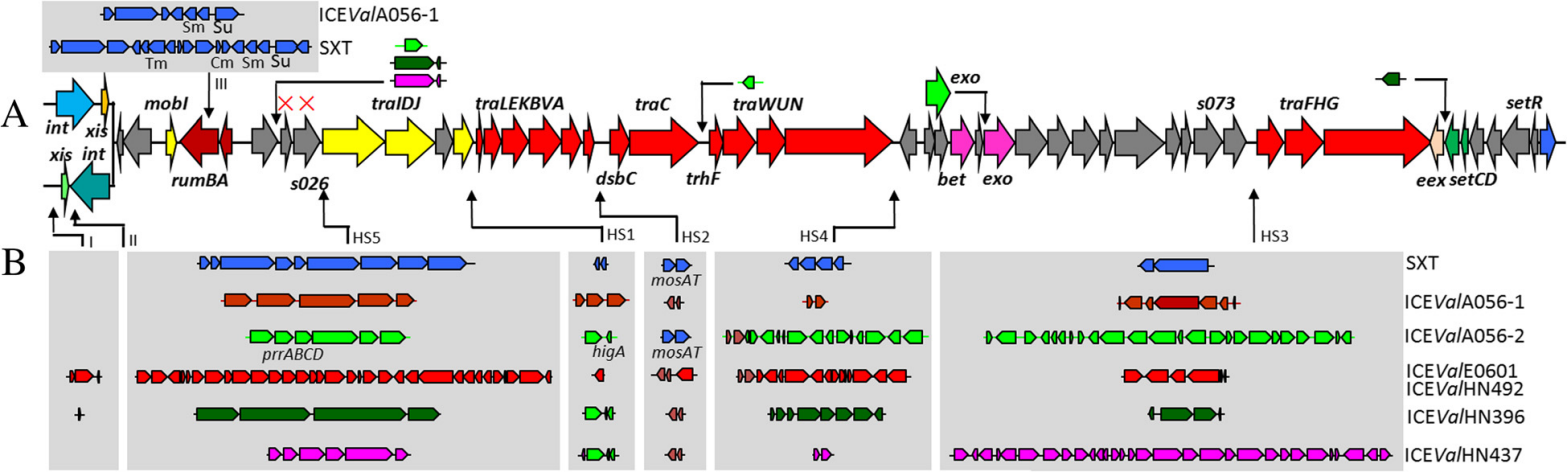
Structural comparison between SXT and six ICEs from *V. alginolyticus*. **a** Schematic representation of the 52 core genes of SXT/R391 ICEs. Left lower part is the normal *int*/*xis* module of SXT/R391 ICEs, and the upper left part shows the alternative *int*/*xis* modules of ICE*Val*A056-2, ICE*Val*HN396 and ICE*Val*HN437. Atypical insertions of ORFs of these ICEs and an extra copy of *exo* gene (*pale green*) of ICE*Val*A056-2 are indicated with fold-down arrows. The absences of *s025* and *s026* in ICE*Val*E0601/ICE*Val*HN492 are shown with red crosses. **b** Variable ICE regions are shown with colors according to the elements in which they were originally described: SXT (*blue*), ICE*Val*A056-1 (*dark red*), ICE*Val*A056-2 (*pale green*), ICE*Val*E0601/ICE*Val*HN492 (*red*), ICE*Val*HN396 (*dark green*) and ICE*Val*HN437 (*pink*). Thin arrows indicate the sites of insertion for each VR region (I, II and III) and HS1–HS5 represent hotspots 1–5. Some notable genes in these regions are marked. Tm, trimethoprim cassette; Cm, chloramphenicol cassette; Sm, streptomycin cassette; Su, sulfamethoxazole cassette; *mosAT*, toxin-antitoxin system; *prrABCD*, an novel operon including a RM system and anticodon nuclease gene. Variable region III is shown at the top of the backbone of SXT/R391 ICEs for saving space

The genomes of six ICEs were analyzed and compared in this study (Table 1). It is very rare to find two ICEs (ICE*Val*A056-1 and ICE*Val*A056-2) coexisting in one strain (*V. alginolyticus* A056). The entire length of ICE*Val*A056-1 is 83.4 Kb, coding for 83 open reading frames (ORFs). Among them, 64 ORFs are present in SXT and 67 ORFs in ICE*Vfl*ind1 (Additional file 1: Table S1). ICE*Val*A056-1 contains a syntenous set of 52 conserved core genes in the SXT/R391 ICEs (Fig. 1). Similar to SXT, specific genes of ICE*Val*A056-1 are inserted into five hotspots (HS1-HS5) and a VR III region, thereby disrupting *rumB* (Fig. 1). The largest variable DNA insertions are 10.0 and 12.4 Kb, occurring in the VR III and HS5 regions, respectively. ICE*Val*A056-2 has a size of 103.8 Kb, including 108 ORFs (Additional file 2: Table S2). Of the 108 ORFs, 54 are present in SXT, ICE*Vch*Mex1 or ICE*Vch*Ban8 (an atypical SXT/R391-related ICE), respectively. The largest variable DNAs (22.3 and 12.4 Kb) are inserted into HS3 and HS4, respectively.

A sequence analysis showed only 14 variable bases dispersed between ICE*Val*E0601 (106.2 Kb) and ICE*Val*HN492 (106.2 Kb) and that all of the genes of ICE*Val*E0601 code amino acid sequences that are identical with the counterparts in ICE*Val*HN492. Therefore, ICE*Val*E0601 and ICE*Val*HN492 were treated as the same ICE. Each of ICE*Val*E0601 and ICE*Val*HN492 contains 113 open ORFs (Additional file 3: Table S3). Among them, 52 ORFs are present in SXT or ICE*Vch*Ind4. Variable DNAs of ICE*Val*E0601 or ICE*Val*HN492 are 54.8 Kb in length, which are distributed between five hotspots (HS1-HS5) and VR I (Fig. 1). The largest insertions of variable DNAs are 28.8 Kb and 12.1 Kb, occurring in the HS5 and HS4 regions, respectively.

ICE*Val*HN396 (86.7 Kb) includes 79 ORFs (Additional file 4: Table S4), of which 51 and 54 ORFs are present in SXT and ICE*Vch*ICDC-1307, respectively. Variable DNAs of ICE*Val*HN396 with a total size of 31.0 Kb are distributed in five HS regions and the VR I region. The largest variable DNAs are 16.5 and 7.5 Kb and are located in the HS5 and HS4 regions, respectively. ICE*Val*HN437 (94.3 Kb) contains 100 ORFs (Additional file 5: Table S5), of which 51 and 52 ORFs are present in SXT and ICE*Vch*Ban9, respectively. Variable DNAs of ICE*Val*HN437 have a total size of 40.1 Kb, which are distributed in five HS regions. The largest variable DNAs are 28.2 and 8.9 Kb, occurring in the HS3 and HS5 regions, respectively.

### Core genes of the ICEs from *V. alginolyticus*

ICE*Val*A056-1 contains a syntenous set of 52 conserved core genes in the SXT/R391 ICEs (Fig. 1 and Additional file 1: Table S1), which are responsible for machinery involved in conjugative transfer, integration/excision and their regulation. Either ICE*Val*E0601 or ICE*Val*HN492 contain 50 out of 52 conserved core genes in the SXT/R391 ICEs (with the exception of genes *s025* and *s026*, which code for predicted proteins of unknown function) (Fig. 1, Additional file 3: Table S3 and Additional file 4: Table S4). S025 and S026 from ICE*Va*lA056-1, ICE*Val*A056-2, ICE*Val*HN396 and ICE*Val*HN437 have low identity values (<90 %) with their counterparts from SXT. ICE*Val*A056-1, ICE*Val*E0601 and ICE*Val*HN492 integrate into the 5′ end of the host’s *prfC* gene targeted by SXT/R391 ICEs [6], and their Int and Xis share at least 97 % identity with their counterparts from SXT.

**Fig. 2:**
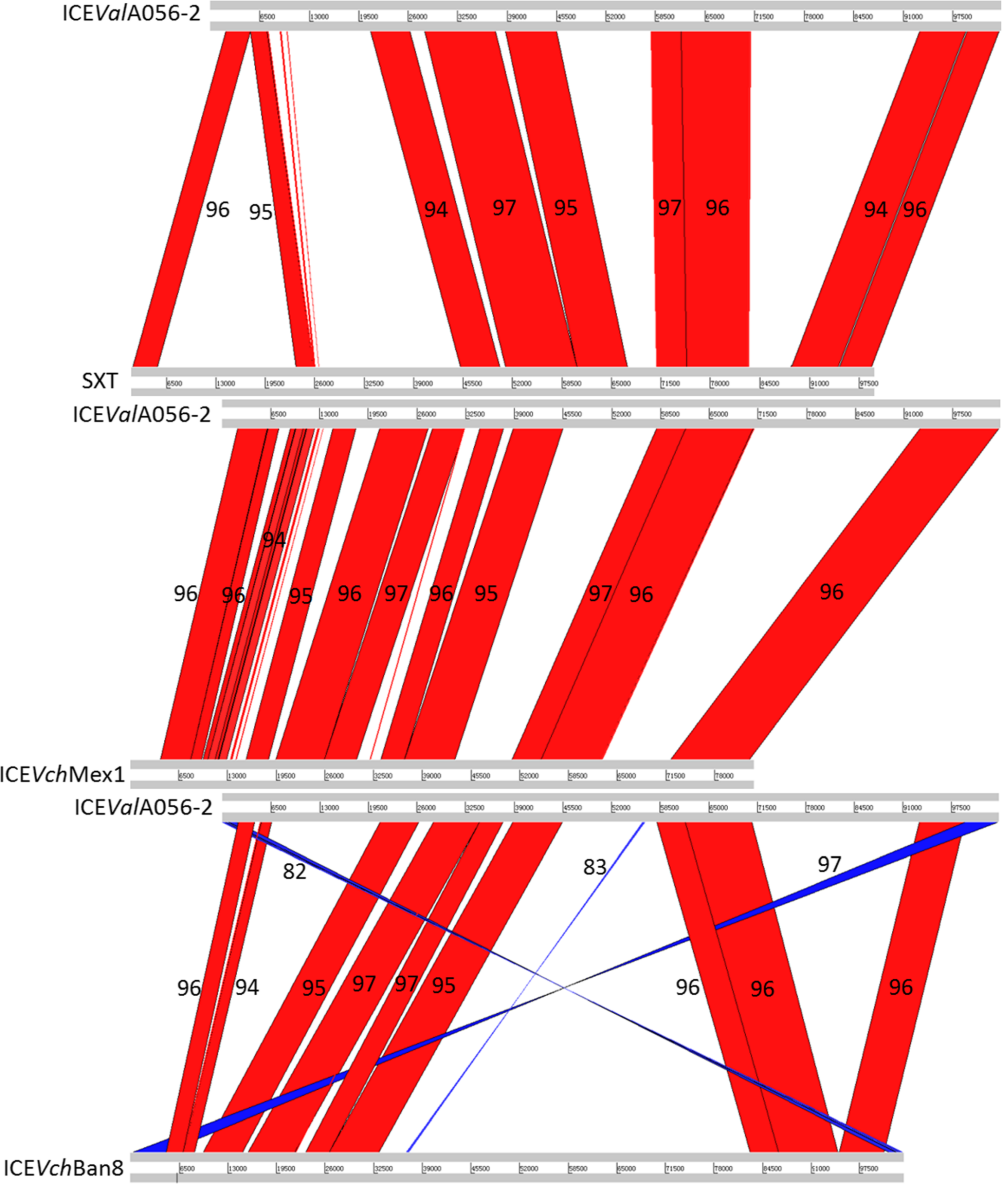
Alignment generated using WebACT for ICE*Val*A056-2, SXT, ICE*Vch*Mex1 and ICE*Vch*Ban8. Comparisons between the regions < 80 bp are filtered. Numbers show the identity values of the compared regions. Red areas indicate homologous regions; blue areas indicate inversions

ICE*Val*A056-2, ICE*Val*HN396 and ICE*Val*HN437 integrate into 3′ end of the *tRNA-ser* gene (Table 1). This locus was reported to be most commonly targeted by genomic islands or by ICE*Vch*Ban8 from a clinical non-toxigenic isolate *V. cholerae* O37 MZO-3 [24, 25]. *int* and *xis* genes of three ICEs locate on the same strand, whereas *int* and *xis* genes of SXT/R391 ICEs locate on different strands. Besides, the two genes lack similarity with their counterparts in the SXT/R391 ICEs. However, when the three ICEs are compared with ICE*Vch*Ban8, there are three obvious differences between two of the ICE types: (1) *int* genes of ICE*Val*A056-2, ICE*Val*HN396 and ICE*Val*HN437 only have 90 % identity with the *int*_*Ban8*_ on the amino acids level, which is substantially lower than the 98 % average identity among SXT/R391 ICEs; (2) *int* followed by *xis* locates at the 5′ end of three ICEs, whereas *int* and *xis* of ICE*Vch*Ban8 locate at the 3′ end of the ICE; and (3) *rumB* genes of three ICEs remain intact without any insertion, but ICE*Vch*Ban8 has two parts of a truncated *rumB* gene. BLASTN searches using the complete sequences of ICE*Val*A056-2, ICE*Val*HN396 and ICE*Val*HN437 as queries also indicated that the most closely related ICEs were ICE*Vch*Mex1, ICE*Vch*ICDC-1307 and ICE*Vch*Ban9, respectively. These sequences are members of the SXT/R391 ICEs. To provide a detailed description of the similarities and differences between the three aforementioned ICEs and their closely related ICEs, representative SXT and ICE*Vch*Ban8, whole-sequence comparisons of these ICEs were performed using WebACT [26]. The highly similar areas (the same orientation, red) between ICE*Val*A056-2 and ICE*Vch*Mex1 (55.7 Kb) or between ICE*Val*A056-2 and SXT (50.1 Kb) are substantially larger than those between ICE*Val*A056-2 and ICE*Vch*Ban8 (43.8 Kb) (Fig. 2). Similarity values between ICE*Val*A056-2 and ICE*Vch*Mex1 (red areas) are generally higher than those between ICE*Val*A056-2 and SXT or ICE*Vch*Ban8 (Fig. 2). Three inversion areas of ICE*Vch*Ban8 clearly highlight major rearrangements in it (blue, Fig. 2). Sequence comparisons between ICE*Val*HN396 and SXT, ICE*Vch*ICDC-1307 (a member of SXT/R391) or ICE*Vch*Ban8 and the comparisons between ICE*Val*HN437 and SXT, ICE*Vch*Ban9 or ICE*Vch*Ban8 also exhibit similar profiles with the comparison between ICE*Val*A056-2 and related ICEs (Additional file 6: Figure S1 and Additional file 7: Figure S2). Therefore, the analysis demonstrated that ICE*Val*A056-2, ICE*Val*HN396 and ICE*Val*HN437 were more similar to genuine SXT/391 ICEs than to ICE*Vch*Ban8, with the exception of the *int*/*xis* module.

**Fig. 3:**
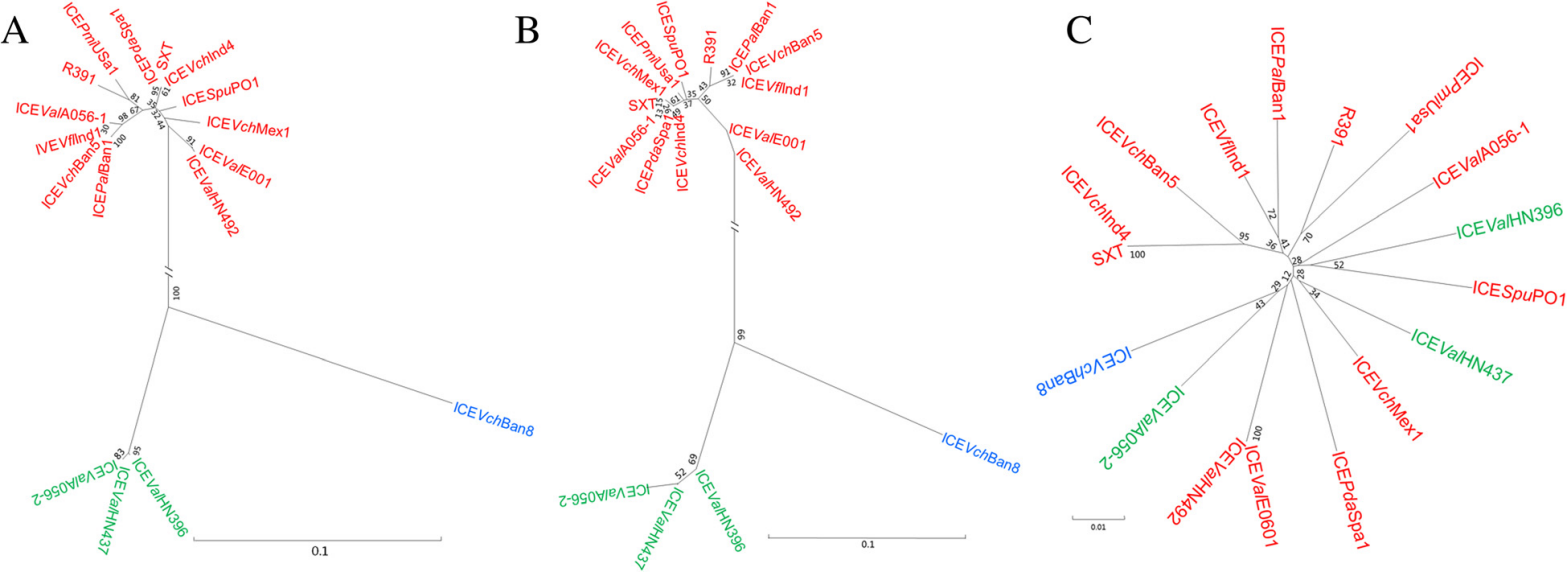
Phylogenetic trees of *int* (**a**), *xis* (**b**) and *traI* (**c**) genes from six ICEs, some randomly selected SXT/R391 ICEs and ICE*Vch*Ban8. The trees were constructed using the neighbor-joining method. Bootstrap values were obtained after 1000 repetitions

To further demonstrate differences in the *int*/*xis* modules contributed by ICE*Val*A056-2, ICE*Val*HN396 and ICE*Val*HN437, we created phylogenetic trees based on respective nucleotide sequences to clarify their evolution. *traI*, one of most divergent core genes in SXT/R391 ICEs [6], was used as a reference gene. The trees for *int* and *xis* were extremely similar (Fig. 3a and b). In both trees, the ICEs were segregated into two evolutionarily distinct groups, whereas *traI* exhibited a completely different branching pattern in which all of the ICEs could not be clustered into different groups (Fig. 3c). In the trees for *int* and *xis*, though four ICEs were grouped into the same big branch, ICE*Vch*Ban8 in each tree formed an independent clade that was much distant from the clade comprised of ICE*Val*A056-2, ICE*Val*HN396 and ICE*Val*HN437. ICE*Val*A056-2, ICE*Val*HN396 and ICE*Val*HN437 have a very similar core genetic structure; however, each has very different variable DNAs (Fig. 2, Additional file 2: Table S2, Additional file 4: Table S4 and Additional file 5: Table S5), and the strains containing ICE*Val*A056-2, ICE*Val*HN396 and ICE*Val*HN437 were isolated in different time and location. Further data mining in GenBank using their *int* genes as queries indicated that *V. vulnificus* CladeA-yb158 (LBNN01000013.1) also potentially contained a complete ICE that was similar to the three ICEs. These results strongly suggest that the occurrence of ICE*Val*A056-2, ICE*Val*HN396 and ICE*Val*HN437 is not a rare event.

Five ICEs (with the exception of ICE*Val*A056-1) in this study maintain intact *rumB* genes without disruption by one typical transposon carrying drug-resistant cassettes. Moreover, it is unusual that ICE*Val*A056-2 contains another version of the *exo* gene located upstream of a standard *exo* gene (Fig. 1). The two *exo* genes share 99 % similarity at the amino acid level.

### Usual gene contents of the hotspots and variable regions of the ICEs in *V. alginolyticus*

The variable genes of six ICEs encode a large array of functions to bestow various adaptive abilities upon their hosts (Table 1, Additional file 1: Tables S1, Additional file 2: Table S2, Additional file 3: Table S3, Additional file 4: Table S4 and Additional file 5: Table S5). A substantial proportion of variable genes have orthologous genes and functionally related genes found in SXT/R391 ICEs, which confer functions typically found in other ICEs. For instances, *mosA*/*T*, *hipA*/*B* and sole *higA* (antitoxin gene to *higB*) encode for toxin-antitoxin (TA) systems that promote ICE maintenance by killing or severely inhibiting the growth of cells that have lost the element [6, 11]. Many genes encode for diverse restriction-modification (RM) systems, helicases and endonucleases. ICE*Val*A056-1, ICE*Val*E0601 and ICE*Val*HN396 contain the genes coding diguanylate cyclase, which are also found in ICE*Vch*Mex1, ICE*Vfl*Ind1 and ICE*Vch*Moz3 [12]. These genes are involved in biofilm formation, motility and virulence in several organisms [27, 28]. Five ICEs with the exception of ICE*Val*A056-2 contain an acetyltransferase gene that is always flanked by a conserved hypothetical gene in HS2 (Fig. 2), and similar genes were only found in ICE*Vch*Mex1 and R391. ICE*Val*A056-1 has a truncated copy of a drug-resistant gene cluster in VR III region that is owned by SXT. Similar truncations were also observed in other ICEs such as ICE*Pal*Ban1 and ICE*Vch*Ind5 [6]. When BLASTN was performed with the whole sequences of any variable DNA regions in ICE*Val*A056-1, highly similar DNA sequences (>98 %) could be found in other ICEs (Additional file 1: Table S1). However, this feature is not obvious in the other five ICEs.

### Unique gene contents of the hotspots and variable regions in the ICEs of *V. alginolyticus*

With the exception of the abovementioned usual genes in hotspots and variable regions shared by other ICEs, six ICEs also contain a large number of unique genes or gene clusters that were not found in other ICEs (Table 1, Additional file 1: Tables S1, Additional file 2: Table S2, Additional file 3: Table S3, Additional file 4: Table S4 and Additional file 5: Table S5). Here, we only analyzed some of their notable genes. A large number of variable genes of ICE*Val*A056-2 code for proteins that are involved in metabolism and transport. The functions of these proteins include choline uptake, the stabilization of the MscS mechano sensitive channel, calcium/sodium proton antiport and alternative metabolic pathways for oxaloacetate and citrate. A novel intact *prrABCD* operon-like structure is present in the HS5 region of ICE*Val*A056-2 (Fig. 1). No similar continuous structure was found in the other bacterial genomes according to a BLASTN search. *prrA*, *prrB* and *prrD* code for a type I DNA RM system that is similar to *hsdMSR* in *Escherichia coli* [29], whereas *prrC* codes for a putative anticodon nuclease. A related *prrABCD* system has been reported in *E. coli* CTr5X [30, 31]. PrrA, PrrB and PrrC of CTr5X only share 82, 33 and 74 % similarity with their counterparts in ICE*Val*A056-2, respectively, whereas the PrrD lacks similarity with the PrrD encoded by ICE*Val*A056-2. Overall, most of the variable genes in ICE*Val*A056-2 could not be found in any other ICEs. Two genes coding for transposases were found to insert in two atypical sites (between *s024* and *s025* and between *traC* and *trhF*) (Fig. 1).

ICE*Val*E0601 bears a large gene cluster composed of at least 22 genes (14.7 Kb) coding for a complex pilus assembly system. A putative threonine efflux gene and a related gene coding hydroxyphenylpyruvate dioxygenase were discovered in ICE*Val*E0601, which were predicted to contribute to amino acid transport and metabolism. A gene coding for MscS mechanosensitive channel protein and a homologous gene to *yrbG* coding for calcium/sodium proton antiporter occur in ICE*Val*E0601, which were predicted to participate in the regulation of osmotic homeostasis protecting the cell from acute decreases in the osmotic environment. ICE*Val*E0601 contains two dissimilar genes that respectively encode for RNA-dependent DNA polymerase participating in DNA replication, recombination and repair.

ICE*Val*HN396 includes a gene cluster composed of at least six genes. These genes code for a bacterial chemotaxis apparatus that enables the bacteria to direct their movement away from unfavorable chemical stimuli and towards favorable chemical compounds [32, 33]. ICE*Val*HN396 contains the gene *dprA* and accessory gene *recQ*. Both ICE*Val*HN396 and IC*Val*HN437 have an atypical insertion of a small hypothetical gene flanked by a transposase gene between *s024* and *s025* (Fig. 1). In addition to this, ICE*Val*HN396 includes another atypical insertion of a gene coding DDE endonuclease between *eex* and *setC* (Fig. 1).

The largest insertion (with a size of 28.2 Kb) occurs in the HS3 region of ICE*Val*HN437 and consists of 30 genes with various functions. The HS3 region contains five known genes coding for UDP-glucose dehydrogenase, UTP-glucose-1-phosphate uridylyltransferase, alpha-L-Rha alpha-1,3-L- rhamnosyltransferase, glycosyl transferase and capsular polysaccharide ABC transporter, respectively. All five genes are involved in the metabolism of nucleotide and carbohydrate and synthesis of capsular polysaccharide. Five known genes are adjoined and dispersed by eight hypothetical genes, among which four hypothetical genes lack homologous genes in GenBank. Based on these features, eight hypothetical genes may be related to nucleotide and carbohydrate metabolism and the synthesis of capsular polysaccharide. The HS3 region also includes a gene cluster comprised of five genes coding for sulfate permease, sulfate adenylyltransferase subunit 2, adenylylsulfate kinase, acetyltransferase and sulfate adenylyltransferase subunit, respectively. Thus, this gene cluster is likely responsible for the metabolic assimilation of sulfur from inorganic sulfate. A sole gene (*osmC*) is coupled by two transposase genes to form a typical transposon structure. *osmC* codes for organic hydroperoxide resistance protein; this gene thus likely confers its host with an alternative function to regulate oxidative stress. The genes similar to *osmC* have never previously been found in *Vibrio* species.

### Intensive distribution of transposase genes in the ICEs from *V. alginolyticus*

Notably, the ICEs from these *V. alginolyticus* strains contain abundant transposase genes (Additional file 1: Tables S1, Additional file 2: Table S2, Additional file 3: Table S3, Additional file 4: Table S4 and Additional file 5: Table S5), with an average of over 7 transposase genes per ICE. A similar phenomenon was observed in other SXT/R391 ICEs, such as SXT, ICE*Vch*Ban5, ICE*Vfl*Ind1 and ICE*Pda*Spa1. The annotations for genomic scaffold sequences of five strains, A056, E0601, HN396, HN437 and HN492, showed that they contained 38, 50, 32, 48 and 43 transposase genes, respectively. The construction of a phylogenetic tree based on the transposase genes in these ICEs failed because they are so highly distinct that no common sites can be found. Blast searches showed that the genes closely matching these distinct transposase genes have extensive sources.

## Discussion

SXT/R391 ICEs widely distribute in various marine-sourced bacteria around the world [34]; however, there were no records indicating the emergence of any ICEs in China until we confirmed the presence of SXT/R391-like ICEs in some *V. alginolyticus* strains in 2012 [19]. To fully understand the features and functions of ICEs in *V. alginolyticus*, we acquired complete sequences of six ICEs in five randomly selected *V. alginolyticus* strains. Strains E0601 and HN492 contain extremely similar ICEs (ICE*Val*E0601 and ICE*Val*HN492) that were isolated from various places (linear distance between two sites is nearly 500 km) in 2006 and 2008, respectively. Similar situations have been observed in the strains containing highly similar SXT_MO10_ and ICE*Vch*Ind4 and the strains containing extremely similar ICE*Vch*Ban9 and ICE*Vch*Moz10; however, these were isolated at distant places and in different years [6]. With the exception of extreme similarity between ICE*Val*E0601 and ICE*Val*HN492, a high genetic diversity between strains HN492 and E0601 was exhibited through their genomic comparison (data not shown), which indicated that they are different strains. These results strengthened the speculation by Wozniak et al. that some ICEs have recently spread [6] because they have not demonstrated obvious divergent evolution.

Regarding the core genes of the six ICEs, ICE*Val*E0601 and ICE*Val*HN492 lack *s025* and *s026.* In another case, the absence of the core genes *s024*, *s025* and *s026* in the atypical element ICE*Vch*Ban8 has been observed [24]. Additionally, S025 and S026 from these ICEs have relatively low identity values with their counterparts from SXT. The significant divergence of S026 had been analyzed; however, S025 has not [6]. Beaber et al. showed that the deletion of five cores gene from *rumB* to *s026* (including *s024* and *s025*) had no detectable influence on SXT excision or transfer [20]. These results indicated that though most SXT/R391- or SXT/R391-related ICEs have the core genes *s024*, *s025* and *s026*, they are not necessary for the maintenance and transfer of ICEs. Thus, these genes can bear more variation due to spontaneous mutations and are potentially lost during recombination events.

ICE*Val*A056-1, ICE*Val*E0601 and ICE*Val*HN492 integrate into the 5′ end of the host’s *prfC* gene targeted by SXT/R391 ICEs [6], and their Int and Xis share at least 97 % identity with their counterparts from SXT. Together with the fact that they share 48 other conserved core genes (with the exception of *s025* and *s026*), these three ICEs are easily classified into the SXT/R391 family based on the strict definition of SXT/R391 ICEs [34].

The classification of ICE*Val*A056-2, ICE*Val*HN396 and ICE*Val*HN437 presents difficulties because they exhibit a different *int*/*xis* module from that in SXT/R391 ICEs and because they integrate into another chromosomal site, the *tRNA-ser* gene. However, comparative genomics demonstrated that ICE*Val*A056-2, ICE*Val*HN396 and ICE*Val*HN437 were more similar to genuine SXT/391 ICEs than to ICE*Vch*Ban8 with the exception of the *int*/*xis* module. A phylogenetic analysis showed that though *int* and *xis* of ICE*Vch*Ban8 were grouped into the same large branch, they formed a very independent clade in the trees of *int* and *xis*. These results clearly show that ICE*Val*A056-2, ICE*Val*HN396 and ICE*Val*HN437 form a group that is independent from ICE*Vch*Ban8. However, these three ICEs cannot be classed into a new ICE family only based on the different *int*/*xis* module and integration site because they retain 50 of 52 conserved core genes in the same organization, which are highly similar to those in SXT/R391 ICEs (with the exception of *s025* and *s026*). *traI is* one of the most divergent core genes in SXT/R391 ICEs [6] and is theoretically more likely to cluster these ICEs into different groups; however, the opposite is true. Based on these facts and other considerations, we prefer to classify ICE*Val*A056-2, ICE*Val*HN396 and ICE*Val*HN43 into an atypical group of SXT/R391 ICEs. This classification will enrich our knowledge to SXT/R391 ICEs and increase the diversity of SXT/R391 ICEs. Because they are the members of an atypical group in SXT/R391 ICEs, it is interesting to speculate on how these atypical ICEs were generated. ICEs have three discrete functional modules that govern integration/excision, conjugative transfer (DNA processing and mating pair formation) and regulation [2, 3]. Therefore, the primary *int*/*xis* module as a whole was likely replaced by the current *int*/*xis* module; primary ICEs could have also been replaced by SXT/R391 ICEs, leaving only *int*/*xis* modules through potential recombination. The latter appears more reasonable because three ICEs integrate into the *tRNA-ser* site that SXT/R391 ICEs never inserts into. Inter-ICE recombination was observed between tandem SXT/R391 ICEs, yielding hybrid ICEs with considerable frequency [35]. Comparative genomics also supports the assertion that inter-ICE recombination is commonplace [6].

Our results revealed that the ICEs analyzed in environmental strains generally retain intact *rumB* genes. The *rumB* and adjacent *rumA* genes encode a UN repair DNA polymerase and a UV repair protein, respectively [36]. Sunlight-emitted UV light represents a major source of genotoxic stress to bacteria in the environment [37]; they therefore tend to conserve ICEs devoid of antibiotic resistance genes by retaining a functional *rumBA* compared with clinical strains not exposed to UV but to antibiotics [38]. Among the six ICEs from *V. alginolyticus*, only ICE*Val*A056-1 contains drug-resistant genes carrying a transposon. Similar results were also found in another report in which only 3 of 11 environmental *Vibrio* strains containing ICEs exhibited resistance to sulfamethoxazole and streptomycin, the typical resistance carried on SXT [9]. Additionally, most drug-resistant cassettes in ICEs are carried by transposons, which increases the likelihood that these drug-resistant cassettes were captured after the acquisition of the ICEs by primary hosts. These results demonstrated that drug resistance was not a necessary function of ICEs, especially in environmental bacteria. However, nearly all of the ICEs discovered contain diverse RM systems, helicases and endonucleases, which likely protect hosts from invasion by foreign DNA (including phage infection) and/or promote the integrity of the ICE genome during its transfer between hosts [6]. Thus, the initial biological significance of ICEs to hosts is to provide protection from invasion by phages and plasmids and even from DNA lesions due to environmental factors.

A sequence analysis of VR and HS regions of six ICEs indicated that unbalanced insertions of variable DNAs are very common. For instance, the HS3 region of ICE*Val*HN437 occupies 70 % of variable DNAs. ICE*Vch*Ban8 has a similar situation, and nearly all variable DNAs are distributed in its HS4 region [24]. Though five hotspots of the ICEs in *V. alginolyticus* strains have variable sizes, the insertional sizes in HS1 never exceed 4 Kb and remain relatively stable. A similar phenomenon was observed in 12 out of 13 typical SXT/R391 ICEs with the exception of ICE*Vfl*Ind1 [6]. Regarding the contents of variable genes, the six ICEs also contain a larger number of variable genes that confer various functions to their hosts. Here, we only discuss some unique genes discovered in these six ICEs. ICE*Val*E0601 bears a large gene cluster coding for a complex pilus assembly system. Pili are involved in adhesion to host cells, motility and DNA exchange [39, 40], and pili are often crucial virulence factors in pathogenic bacteria because they mediate attachment to and infection of target cells and are involved in evasion of the host immune system [41]. ICE*Val*HN396 contains the gene *dprA*, which codes for DNA recombination mediator protein A. This gene is predicted to bind cooperatively to single-stranded DNA (ssDNA) and to interact with RecA [42, 43]. In the process, DprA-RecA-ssDNA filaments are produced; these filaments catalyze the homology-dependent formation of joint molecules [42, 43]. DprA is a new member of the recombination-mediator protein family and performs natural bacterial transformation [42–44]. This indicates that ICEs not only increase the genetic diversity of their hosts in their integration but also have the ability to facilitate bacterial gene exchange via natural bacterial transformation. Many alternatively metabolic pathways were discovered in six ICEs. As a member of bacterioplankton, *V. alginolyticus* widely distributes in various marine environments through ocean currents. Increased metabolic pathways could potentially strengthen the fitness of *V. alginolyticus* in various environments.

In our study, abundant transposase genes were discovered in six ICEs from *V. alginolyticus* and other ICEs. Transposase genes are the most abundant genes in both completely sequenced genomes and environmental metagenomes and are also the most ubiquitous in metagenomes [45, 46]. Previous statistics from 630 bacterial genomes by Aziz et al. also showed that the average number of transposase genes in bacteria was 40 per genome [46]. Considering this average value and the size of the ICEs (generally less than 150 Kb), transposase genes may have a more intensive distribution in these ICEs than in any other parts of their host genomes. Transposase genes encode DNA-binding enzymes that catalyze ‘cut-and-paste’ or ‘copy-and-paste’ reactions to promote the integration of DNA segments into new sites [47]. Most of the described transposase genes are the core parts of transposable genetic elements (TEs), consisting of insertion sequence (IS) elements and their resulting transposon elements (Tn), which play an important role in bacterial fitness and evolution through horizontal gene transfer [48–50]. Based on these analyses, these transposase genes likely integrated into the hotspots or variable regions of ICEs after the creation of the ICEs. The acquisition of TEs does not always benefit hosts; TEs can exhibit detrimental effects by inactivating housekeeping genes or impairing the chromosome’s structure due to transposition to unsuitable sites [26, 48, 51, 52]. The accommodation of various TEs into one relatively unimportant genomic locus exhibits fewer hazards than that into multiple dispersed loci. ICEs can represent an ideal target for the ‘foothold’ of these TEs due to their insertional sites and containing capability. This may explain why transposase genes occur intensively in ICEs. ICEs have sophisticated transfer machinery and can transfer between genetically distant species at a relatively high frequency of 10^−5^-10^−6^ per donor or recipient [7, 20, 22, 53]; consequently, ICEs may serve as a carrier for TEs and largely facilitate the dissemination of TEs.

## Conclusions

In this study, we acquired complete sequences for six new ICEs in *V. alginolyticus* strains. A comparative genomic analysis showed that ICE*Val*A056-1, ICE*Val*E0601 and ICE*Val*HN492 are typical members of the SXT/R391 family. ICE*Val*A056-2, ICE*Val*HN396 and ICE*Val*HN437 represent an atypical group in SXT/R391 family. The variable genes of six ICEs encode a large array of functions to bestow various adaptive abilities upon their hosts. Many variable genes have orthologous and functionally related genes found in SXT/R391 ICEs. Six ICEs also contain a large number of unique genes or gene clusters that were not found in other ICEs. Diverse transposase genes intensively occur in the ICEs, and ICEs may serve as a carrier for TEs and largely facilitate the dissemination of TEs.

## Methods

### Genome sequencing and assemblies of six ICEs from *V. alginolyticus* strains

Five *V. alginolyticus* strains, A056, E0601, HN396, HN437 and HN492 (their sources are listed in Table 1), were sequenced on the HiSeq 2000 sequencing platform (BGI, China) with a paired-end 2 × 100-nucleotide (nt) procedure. *De novo* assemblies were performed through the CLC Genomics Work Bench and SeqMan [54].

Genome sequences of *V. alginolyticus* strains were annotated using the RAST annotation pipeline [23]. The scaffolds containing fragments of ICE elements were singled out and aligned manually with ICE sequences of SXT (AY055428.1), ICE*Vch*Ind4 (GQ463141.1) and ICE*Vch*Mex1 (GQ463143.1) as references. Gaps between two scaffolds were closed using manual editing via PCR amplification and sequencing.

### PCR conditions

PCR primers are listed in Additional file 8: Table S6. All PCRs for gap filling were performed in a 50-μl reaction containing 1 μl of genomic DNA, 0.4 μM of each primer, 5 μl of 10 × PCR buffer, 0.2 mM dNTP and 1 U of Taq DNA polymerase (Takara, China). The amplification program consisted of an initial denaturation at 94 °C for 4 min, 30 cycles of denaturation at 94 °C for 20 s, annealing at 54–58 °C for 30 s, and extension at 72 °C for 1–2 min with a final extension at 72 °C for 7 min.

### Bioinformatics

Related ICEs sequences were searched via BLASTN using the entire sequence of each ICE as a query. The ICE*Val*A056-2, ICE*Val*HN396 and ICE*Val*HN437 sequences were compared with related ICE sequences using WebACT [26]. Similarities in nucleotide and protein sequences for two ICEs were determined as the % nucleotide or amino acid identity with related ICEs by employing BLASTN and BLASTP. The genes analyzed were aligned using ClustalW in MEGA 6.0, and phylogenetic trees were constructed using the neighbor-joining method in MEGA 6.0. The reliability of each tree was subjected to a bootstrap test of 1000 repetitions.

## Additional files

Additional file 1: Table S1. ORFs in ICE*Val*A056-1 and their similarity with related ICEs. (DOCX 21 kb) Additional file 2: Table S2. ORFs in ICE*Val*A056-2 and their similarity with related ICEs. (DOCX 22 kb) Additional file 3: Table S3. ORFs in ICE*Val*E0601 (ICE*Val*HN492) and their similarity with related ICEs. (DOCX 21 kb) Additional file 4: Table S4. ORFs in ICE*Val*HN396 and their similarity with related ICEs. (DOCX 19 kb) Additional file 5: Table S5. ORFs in ICE*Val*HN437 and their similarity with related ICEs (DOCX 19 kb) Additional file 6: Figure S1. Alignment generated using WebACT of ICE*Val*HN396, SXT, ICE*Vch*ICDC-1307 and ICE*Vch*Ban8. Comparisons between the regions < 80 bp are filtered. Numbers show the identity values of the compared regions. Red areas indicate homologous regions; blue areas indicate inversions. (TIF 522 kb) Additional file 7: Figure S2. Alignment generated using WebACT of ICE*Val*HN437, SXT, ICE*Vch*Ban9 and ICE*Vch*Ban8. Comparisons between the regions < 80 bp are filtered. Numbers show the identity values of the compared regions. Red areas indicate homologous regions; blue areas indicate inversions. (TIF 493 kb) Additional file 8: Table S6. Primers used for gap filling in this study. (DOCX 15 kb)
