# Supplementary material for: Comparative genomic analysis of six new-found integrative conjugative elements (ICEs) in Vibrio alginolyticus
Source: BMC Microbiol. 2016 May 4;16:79. doi: 10.1186/s12866-016-0692-9 (PMC4857294; doi:10.1186/s12866-016-0692-9)
Supplement: Additional file 1: Table S1. — ORFs in ICEValA056-1 and their similarity with related ICEs. (DOCX 21 kb) [file 12866_2016_692_MOESM1_ESM.docx]

**Additional file 1: Table S1.** ORFs in ICE*Val*A056-1 and their similarity with related ICEs

| Functions of genes^1^ | Length (aa) | % Identity^2^ | | Remarks^3^ |
| --- | --- | --- | --- | --- |
|  |  | SXT | ICEV*fl*Ind1 |  |
| Recombination directionality factor, Xis | 64 | 100 | 95 |  |
| Integrase, Int | 413 | 97 | 98 |  |
| Hypothetical protein | 89 | 96 | 100 |  |
| Rod shape determination protein | 324 | 96 | 100 |  |
| Hypothetical protein | 55 | 96 | 100 |  |
| Hypothetical protein | 44 | 94 | 100 |  |
| Hypothetical protein, MobI | 147 | 97 | 100 |  |
| Error-prone repair protein, RumB | 330 | 95 | 98 |  |
| Transposase | 197 | 100 | 100 | ICE*Vch*VC883 |
| Transposase | 992 | 99 | 100 |  |
| Transposase | 180 | 100 | 100 |  |
| Streptomycin 3'-kinase, StrB | 278 | 100 | 100 |  |
| Streptomycin 3'-kinase, StrA | 267 | 100 | 99 |  |
| Dihydropteroate synthase | 271 | 100 | 99 |  |
| Transposase | 537 | 100 | 99 |  |
| Error-prone repair protein, RumB | 89 | 100 | 100 |  |
| Error-prone repair protein, RumA | 140 | 100 | 99 |  |
| Hypothetical protein | 85 | 100 | 96 |  |
| DNA polymerase III, S024 | 301 | 98 | 97 |  |
| Hypothetical protein, S025 | 99 | 68 | 95 |  |
| Hypothetical protein, S026 | 307 | 57 | 87 |  |
| Type III RM system methylase | 606 | - | - | \| ICE*Val*Spa1 \| \| --- \| |
| Type III RM system helicase | 883 | - | - |  |
| Hypothetical protein | 1293 | - | - |  |
| Hypothetical protein | 806 | - | - |  |
| Putative inner membrane protein | 393 | - | 83 |  |
| TraI | 716 | 93 | 94 |  |
| TraD | 606 | 97 | 95 |  |
| Conjugative transfer protein | 186 | 93 | 94 |  |
| TraJ | 211 | 97 | 96 |  |
| Hypothetical protein | 212 | - | - | ICE*Eni*Spa1 |
| Transcriptional regulator | 368 | - | - |  |
| Mobile element protein | 401 | - | - |  |
| TraL | 93 | 96 | 98 |  |
| TraE | 208 | 96 | 95 |  |
| TraK | 298 | 97 | 94 |  |
| TraB | 429 | 96 | 97 |  |
| TraV | 216 | 98 | 98 |  |
| TraA | 128 | 97 | 97 |  |
| Acetyltransferase | 168 | - | - | R391 |
| Hypothetical protein | 88 | - | - |  |
| DsbC | 230 | 97 | 96 |  |
| TraC | 799 | 96 | 96 |  |
| Conjugative transfer protein | 115 | 97 | 97 |  |
| Conjugative signal peptidase, TrhF | 170 | 96 | 97 |  |
| TraW | 374 | 96 | 97 |  |
| TraU | 326 | 97 | 98 |  |
| TraN | 1230 | 94 | 96 |  |
| Hypothetical protein | 110 | - | 96 |  |
| Hypothetical protein | 220 | - | 99 |  |
| Hypothetical protein | 200 | 97 | 100 |  |
| Hypothetical protein | 108 | 97 | 100 |  |
| Single-stranded DNA-binding protein, Ssb | 139 | 96 | 100 |  |
| Recombination protein, Bet | 272 | 97 | 99 |  |
| hypothetical protein, OrfZ | 47 | 98 | 97 |  |
| Recombination-related exonuclease, Exo | 338 | 98 | 100 |  |
| Aerobic cobaltochelatase, CobS | 319 | 96 | 100 |  |
| Hypothetical protein | 255 | 97 | 100 |  |
| Cobalamine biosynthesis protein | 317 | 97 | 99 |  |
| Hypothetical protein | 146 | 96 | 100 |  |
| Plasmid associated protein | 551 | 96 | 100 |  |
| DNA repair protein, RadC | 165 | 96 | 100 |  |
| Hypothetical protein | 113 | 97 | 100 |  |
| Putative primase | 357 | 95 | 98 |  |
| Hypothetical protein | 235 | 93 | 94 |  |
| Hypothetical protein | 38 | - | - | An unidentified ICE in *Shewanella putrefaciens* 200 |
| Diguanylate cyclase | 381 | - | - |  |
| Hypothetical protein | 158 | - | - |  |
| Acriflavin resistance protein | 1013 | - | - |  |
| Membrane-fusion protein | 376 | - | - |  |
| Transcriptional regulator, TetR | 196 | - | - |  |
| Hypothetical protein | 43 | - | - |  |
| TraF | 314 | 94 | 93 |  |
| TraH | 462 | 96 | 94 |  |
| TraG | 1189 | 96 | 95 |  |
| hypothetical protein, Eex | 143 | 98 | 95 |  |
| Transcriptional activator, SetC | 177 | 97 | 96 |  |
| Transcriptional activator, SetD | 99 | 98 | 96 |  |
| LysM/invasin regulatory protein | 182 | 97 | 97 |  |
| Hypothetical protein | 220 | 97 | 98 |  |
| Hypothetical protein | 289 | 97 | 97 |  |
| Transcriptional repressor, SetQ | 83 | 99 | 100 |  |
| Putative cI prophage repressor protein, SetR | 215 | 98 | 98 |  |

^1^ Contents of five hotspots and a variable region are shown in red

^2^ Amino acid sequences of ORFs are compared for identity analysis

^3^ The whole sequences of each variable DNA fragments were searched through BLASTN
