# Supplementary material for: Comparative genomic analysis of six new-found integrative conjugative elements (ICEs) in Vibrio alginolyticus
Source: BMC Microbiol. 2016 May 4;16:79. doi: 10.1186/s12866-016-0692-9 (PMC4857294; doi:10.1186/s12866-016-0692-9)
Supplement: Additional file 2: Table S2. — ORFs in ICEValA056-2 and their similarity with related ICEs. (DOCX 22 kb) [file 12866_2016_692_MOESM2_ESM.docx]

**Additional file 2: Table S2.** ORFs in ICE*Val*A056-2 and their similarity with related ICEs

| Functions of genes^1^ | Length (aa) | % Identity^2^ | | |
| --- | --- | --- | --- | --- |
|  |  | SXT | ICEVchMex1 | ICE*Vch*Ban8 |
| Phage integrase | 410 | - | - | 90 |
| Recombination directionality factor, Xis | 63 | - | - | 90 |
| Hypothetical protein | 87 | 94 | 88 | 87 |
| Rod shape determination protein | 324 | 99 | 99 | 98 |
| Hypothetical protein | 40 | - | 95 | 90 |
| Hypothetical protein, MobI | 147 | 99 | 99 | 97 |
| Error-prone repair protein, RumB | 422 | 98 | 98 | - |
| Error-prone repair protein, RumA | 149 | 98 | 98 | 95 |
| Hypothetical protein | 159 | - | - | - |
| DNA polymerase III, S024 | 906 | 98 | 98 | - |
| **Transposase^3^** | **1329** | **-** | **-** | **-** |
| Hypothetical protein, S025 | 282 | 73 | 90 | - |
| Hypothetical protein, S026 | 954 | 63 | 92 | - |
| Type I RM system, HsdM^2^ | 521 | - | 95 | - |
| Type I RM system, HsdS | 404 | - | - | - |
| Anticodon nuclease | 391 | - | - | - |
| Type I RM system, HsdR | 1038 | - | - | - |
| Hypothetical protein | 419 | - | - |  |
| Bipolar DNA helicase, HerA | 577 | - | - | - |
| TraI | 716 | 92 | 94 | 93 |
| TraD | 606 | 99 | 99 | 99 |
| Conjugative transfer protein | 186 | 92 | 96 | 94 |
| TraJ | 211 | 99 | 99 | 99 |
| Fic family protein | 368 | - | 99 | - |
| HigA (antitoxin to HigB) | 96 | - | - | - |
| TraL | 93 | 99 | 99 | 100 |
| TraE | 208 | 99 | 99 | 99 |
| TraK | 298 | 97 | 97 | 96 |
| TraB | 429 | 97 | 98 | 99 |
| TraV | 190 | 97 | 98 | 98 |
| TraA | 128 | 100 | 99 | 99 |
| MosT | 277 | 99 | - | 99 |
| MosA | 312 | 100 | - | 99 |
| DsbC | 230 | 99 | 99 | 99 |
| TraC | 799 | 99 | 99 | 99 |
| **Transposase^3^** | **295** | **-** | **-** | **-** |
| Conjugative transfer protein | 87 | 96 | 96 | 95 |
| Conjugative signal peptidase, TrhF | 170 | 96 | 99 | 99 |
| TraW | 374 | 97 | 96 | 96 |
| TraU | 326 | 99 | 99 | 99 |
| TraN | 1230 | 98 | 97 | 97 |
| Hypothetical protein | 110 | - | - | - |
| Hypothetical protein | 229 |  | - |  |
| Mobile element protein | 88 | - | - | 65 |
| Mobile element protein | 142 | - | - | - |
| Transposase | 299 | - | - | - |
| Hypothetical protein | 65 | - | - | - |
| Hypothetical protein | 37 | - | - | - |
| High-affinity choline uptake protein, BetT | 422 | - | - | - |
| Mechanosensitive channel-related protein | 285 | - | - | - |
| Predicted calcium/sodium:proton antiporter | 156 | - | - | - |
| Mobile element protein | 285 | - | - | - |
| Hypothetical protein | 41 | - | - | - |
| Hypothetical protein | 126 | - | - | - |
| Retron-type RNA-directed DNA polymerase | 434 | - | - | - |
| Mobile element protein | 284 | - | - | - |
| Mobile element protein | 443 | - | - | - |
| Hypothetical protein | 200 | 99 | 98 | 91 |
| Hypothetical protein | 108 | 95 | 92 | 93 |
| Single-stranded DNA-binding protein, Ssb | 139 | 96 | 98 | 97 |
| Recombination protein, Bet | 272 | 99 | 99 | 61 |
| Hypothetical protein, OrfZ | 47 | 99 | 99 | 98 |
| **Recombination-related exonuclease, Exo**^4^ | **335** | **99** | **99** | **98** |
| Recombination-related exonuclease, Exo | 338 | 99 | 99 | 99 |
| Aerobic cobaltochelatase, CobS | 258 | 98 | 97 | 98 |
| Hypothetical protein | 255 | 98 | 96 | 97 |
| Cobalamine biosynthesis protein | 317 | 99 | 99 | 99 |
| Hypothetical protein | 146 | 97 | 97 | 95 |
| Plasmid associated protein | 551 | 97 | 97 | 96 |
| DNA repair protein, RadC | 165 | 99 | 99 | 100 |
| Hypothetical protein | 113 | 96 | 99 | 97 |
| Putative primase | 357 | 96 | 96 | 96 |
| Hypothetical protein | 235 | 91 | 91 | 84 |
| Protein of unknown function | 110 | - | - | - |
| Hypothetical protein | 450 | - | - | - |
| Hypothetical protein | 264 | - | - | - |
| Hypothetical protein | 246 | - | - |  |
| Hypothetical protein | 143 | - | - | - |
| DNA repair protein, RadC | 157 |  |  |  |
| Predicted transcriptional regulator | 59 | - | - | - |
| Hypothetical protein | 91 | - | - | - |
| Transcriptional regulatory protein, CitB | 226 | - | - | - |
| Sensor kinase, CitA, | 551 | - |  | - |
| α-Oxaloacetate decarboxylase | 603 | - | - | - |
| β-Oxaloacetate decarboxylase | 433 | - | - | - |
| y-Oxaloacetate decarboxylase | 83 | - | - |  |
| Na-Citrate antiporter | 448 | - | - | - |
| [Citrate [pro-3S]-lyase] ligase | 350 | - | - | - |
| y-Citrate lyase | 98 | - | - | - |
| β-Citrate lyase | 291 | - | - | - |
| α-Citrate lyase | 505 | - | - | - |
| ε-Citrate lyase | 178 | - | - | - |
| CitG | 319 | - | - | - |
| Hypothetical protein | 275 | - | - | - |
| Mobile element protein | 444 | - | - | - |
| Mobile element protein | 86 | - | - | - |
| Mobile element protein | 220 | - | - | - |
| TraF | 310 | 94 | 97 | 98 |
| TraH | 462 | 99 | 98 | 98 |
| TraG | 1189 | 98 | 99 | 98 |
| Hypothetical protein, Eex | 143 | 73 | - | 91 |
| Transcriptional activator, SetC | 177 | 99 | 99 | 99 |
| Transcriptional activator, SetD | 99 | 100 | 98 | 100 |
| LysM/invasin protein | 182 | 98 | 98 | 98 |
| Hypothetical protein | 220 | 99 | 94 | 98 |
| Hypothetical protein | 289 | 98 | 98 | 99 |
| Transcriptional repressor, SetQ | 83 | 100 | 99 | 100 |
| Putative cI prophage repressor protein, setR | 215 | 97 | 97 | 96 |

^1^Contents of five hotspots are shown in red

^2^ Amino acid sequences of ORFs were compared for identity analysis

^3^ Two atypical insertions of transposase genes between the backbone genes are in bold

^4^ Another copy of the gene coding for recombination protein is shown in bold
