# Supplementary material for: Comparative genomic analysis of six new-found integrative conjugative elements (ICEs) in Vibrio alginolyticus
Source: BMC Microbiol. 2016 May 4;16:79. doi: 10.1186/s12866-016-0692-9 (PMC4857294; doi:10.1186/s12866-016-0692-9)
Supplement: Additional file 3: Table S3. — ORFs in ICEValE0601 (ICEValHN492) and their similarity with related ICEs. (DOCX 21 kb) [file 12866_2016_692_MOESM3_ESM.docx]

**Additional file 3: Table S3.** ORFs in ICE*Val*E0601 (ICE*Val*HN492) and their similarity with related ICEs^1^

| Functions of genes^2^ | Length | % Identity^3^ | |
| --- | --- | --- | --- |
|  |  | SXT | ICE*Vch*Ind4 |
| Possible Antitoxin protein, HipB | 106 |  |  |
| Possible toxin protein, HipA | 404 |  |  |
| Hypothetical protein | 37 |  |  |
| Recombination directionality factor, Xis | 64 | 100 | 100 |
| Integrase | 413 | 99 | 99 |
| Hypothetical protein, S002 | 89 | 96 | 96 |
| Rod shape determination protein,S003 | 324 | 97 | 97 |
| Hypothetical protein | 40 | 95 | 95 |
| Hypothetical protein, MobI | 147 | 99 | 99 |
| Error-prone repair protein, RumB | 422 | 97 | 91 |
| Error-prone repair protein, RumA | 149 | 97 | 97 |
| Hypothetical protein | 45 |  |  |
| DNA polymerase III, S024 | 219 | 84 | 84 |
| Transposase | 305 |  |  |
| DDE endonuclease | 346 |  |  |
| Transposase | 273 |  |  |
| Flp pilus assembly protein | 71 |  |  |
| Type IV prepilin peptidase, CpaA | 90 |  |  |
| Flp pilus assembly protein, CpaB | 268 |  |  |
| Type II/IV secretion system protein, CpaC | 442 |  |  |
| Hypothetical protein | 176 |  |  |
| Type II/IV secretion system ATPase, CpaE | 416 |  |  |
| Type II/IV secretion system protein, CpaF | 429 |  |  |
| Flp pilus assembly protein TadB | 306 |  |  |
| Type II/IV secretion system protein, TadC | 302 |  |  |
| Flp pilus assembly protein, TadD | 298 |  |  |
| Flp pilus assembly membrane protein, TadE | 172 |  |  |
| Flp pilus assembly surface protein, TadF | 179 |  |  |
| Flp pilus assembly protei, TadG | 442 |  |  |
| Outer membrane protein | 213 |  |  |
| Hypothetical protein | 49 |  |  |
| Transposase | 312 |  |  |
| Transposase | 306 |  |  |
| Two-component system response regulator | 224 |  |  |
| P pilus assembly protein related protein | 292 |  |  |
| P pilus assembly protein, porin, PapC | 820 |  |  |
| Hypothetical protein | 227 |  |  |
| P pilus assembly protein, PapD | 243 |  |  |
| Hypothetical protein | 164 |  |  |
| Hypothetical protein | 164 |  |  |
| Threonine efflux protein | 211 |  |  |
| Hypothetical protein | 40 |  |  |
| 4-hydroxyphenylpyruvate dioxygenase | 270 |  |  |
| Type III restriction endonuclease | 569 |  |  |
| Hypothetical protein | 100 |  |  |
| TraI | 726 | 93 | 93 |
| TraD | 606 | 99 | 99 |
| Conjugative transfer protein 234 | 186 | 95 | 95 |
| TraJ | 211 | 98 | 98 |
| Hypothetical protein | 195 |  |  |
| TraL | 93 | 99 | 99 |
| TraE | 204 | 99 | 99 |
| TraK | 298 | 97 | 97 |
| TraB | 429 | 98 | 98 |
| TraV | 190 | 97 | 97 |
| TraA | 128 | 99 | 99 |
| Acetyltransferase | 168 |  |  |
| Conserved hypothetical protein | 88 |  |  |
| Transposase | 367 |  |  |
| DsbC | 230 | 99 | 99 |
| TraC | 799 | 99 | 99 |
| Conjugative transfer protein 345 | 115 | 100 | 100 |
| Conjugative signal peptidase, TrhF | 147 | 95 | 95 |
| TraW | 374 | 97 | 97 |
| TraU | 326 | 98 | 98 |
| TraN | 1230 | 97 | 97 |
| Hypothetical protein | 110 |  |  |
| Hypothetical protein | 229 |  |  |
| Transposase | 142 |  |  |
| Transposase | 299 |  |  |
| Hypothetical protein | 100 |  |  |
| High-affinity choline uptake protein, BetT | 547 |  |  |
| Mechanosensitive channel-related protein | 285 |  |  |
| Inner membrane protein, YrbG | 160 |  |  |
| Transposase | 160 |  |  |
| Transposase | 129 |  |  |
| Hypothetical protein | 41 |  |  |
| Hypothetical protein | 69 |  |  |
| RNA-dependent DNA polymerase | 434 |  |  |
| DDE endonuclease | 292 |  |  |
| Transposase | 443 |  |  |
| Hypothetical protein, S063 | 200 | 99 | 99 |
| Hypothetical protein, S089 | 108 | 95 | 95 |
| Single-stranded DNA-binding protein, Ssb | 139 | 96 | 96 |
| Recombination protein, Bet | 272 | 95 | 99 |
| Hypothetical protein, OrfZ | 47 | 100 | 100 |
| Recombination related exonuclease, Exo | 338 | 99 | 99 |
| Aerobic cobaltochelatase, CobS | 319 | 99 | 99 |
| Hypothetical protein | 255 | 99 | 99 |
| Cobalamine biosynthesis protein | 317 | 98 | 98 |
| Hypothetical protein | 146 | 98 | 98 |
| Plasmid associated protein | 551 | 97 | 97 |
| DNA repair protein, RadC | 165 | 99 | 99 |
| Hypothetical protein | 113 | 94 | 94 |
| Putative primase | 357 | 99 | 99 |
| Hypothetical protein | 235 | 93 | 93 |
| Glucose-1-phosphate adenylyltransferase | 418 |  |  |
| DNA polymerase | 490 |  |  |
| Transposase | 333 |  |  |
| Diguanylate cyclase | 732 |  |  |
| Hypothetical protein | 40 |  |  |
| Hypothetical protein | 41 |  |  |
| TraF | 310 | 93 | 93 |
| TraH | 462 | 99 | 99 |
| Exclusion system, TraG | 1189 | 99 | 99 |
| Exclusion system protein, Eex | 143 | 73 | 73 |
| Transcriptional activator, SetC | 177 | 98 | 98 |
| Transcriptional activator, SetD | 99 | 100 | 100 |
| LysM/invasin protein | 182 | 99 | 99 |
| Hypothetical protein, S083 | 220 | 99 | 99 |
| Hypothetical protein, S084 | 289 | 98 | 98 |
| Hypothetical protein, SetQ | 83 | 100 | 100 |
| cI prophage repressor protein, SetR | 215 | 100 | 100 |

^1^ ICE*Val*E0601 and ICE*Val*HN492 are treated as a same ICE

^2^ Contents of five hotspots and variable region I are shown in red

^3^Amino acid sequences of ORFs were compared for identity analysis
