# Supplementary material for: Comparative genomic analysis of six new-found integrative conjugative elements (ICEs) in Vibrio alginolyticus
Source: BMC Microbiol. 2016 May 4;16:79. doi: 10.1186/s12866-016-0692-9 (PMC4857294; doi:10.1186/s12866-016-0692-9)
Supplement: Additional file 4: Table S4. — ORFs in ICEValHN396 and their similarity with related ICEs. (DOCX 19 kb) [file 12866_2016_692_MOESM4_ESM.docx]

**Additional file 4: Table S4.** ORFs in ICE*Val*HN396 and their similarity with related ICEs

| Functions of genes^1^ | Length(aa) | % Identity^2^ | | |
| --- | --- | --- | --- | --- |
|  |  | SXT | | ICDC-1307 |
| Hypothetical protein | 39 |  | |  |
| Integrase, Int | 410 |  | |  |
| Recombination directionality factor, Xis | 63 |  | |  |
| Hypothetical protein, S002 | 90 | 96 | | 97 |
| Rod shape determination protein, S003 | 324 | 99 | | 99 |
| Hypothetical protein | 41 |  | |  |
| Hypothetical protein, MobI | 147 | 99 | 99 | |
| Error-prone repair protein, RumB | 403 | 98 | | 92 |
| Error-prone repair protein, RumA | 149 | 99 | | 99 |
| DNA polymerase III, S024 | 301 | 97 | | 98 |
| **Transposase ^3^** | **459** |  | |  |
| **Hypothetical protein^3^** | **49** |  | |  |
| Hypothetical protein,S025 | 62 | 89 | | 95 |
| Hypothetical protein, S026 | 307 | 62 | | 97 |
| ATP-dependent helicase | 962 |  | | 99 |
| Type II restriction enzyme, methylase subunits | 1623 |  | | 78 |
| DEAD/DEAH box helicase | 2140 |  | | 99 |
| Putative DNA helicase | 711 |  | | 99 |
| TraI | 716 | 93 | | 93 |
| TraD | 611 | 99 | | 98 |
| Conjugative transfer protein 234 | 186 | 95 | | 96 |
| TraJ | 211 | 99 | | 99 |
| Fic family protein | 368 |  | |  |
| Hypothetical protein | 44 |  | |  |
| HigA protein (antitoxin to HigB) | 98 |  | |  |
| TraL | 93 | 99 | | 98 |
| TraE | 208 | 99 | | 100 |
| TraK | 298 | 97 | | 95 |
| TraB | 429 | 98 | | 98 |
| TraV | 216 | 98 | | 98 |
| TraA | 128 | 100 | | 100 |
| Acetyltransferase | 168 |  | |  |
| Conserved hypothetical protein | 88 |  | |  |
| DsbC | 230 | 99 | | 99 |
| TraC | 800 | 99 | | 99 |
| Conjugative transfer protein 345 | 115 | 99 | | 97 |
| TrhF | 170 | 97 | | 98 |
| TraW | 374 | 96 | | 97 |
| TraU | 342 | 99 | | 99 |
| TraN | 1230 | 97 | | 98 |
| Hypothetical protein | 79 |  | |  |
| ATP-binding protein | 197 |  | |  |
| Predicted transcriptional regulator | 134 |  | |  |
| Aerotaxis sensor receptor protein | 516 |  | |  |
| Chemotaxis protein, CheV | 308 |  | |  |
| Diguanylate cyclase | 386 |  | |  |
| Methyl-accepting chemotaxis protein | 429 |  | |  |
| Transposase | 175 |  | |  |
| Hypothetical protein,s063 | 195 | 100 | | 99 |
| Hypothetical protein, S089 | 114 | 92 | | 92 |
| Single-stranded DNA-binding protein, Ssb | 139 | 96 | | 99 |
| Recombination protein, Bet | 272 | 99 | | 99 |
| Hypothetical protein, OrfZ | 47 | 98 | | 98 |
| Recombination related exonuclease, Exo | 338 | 99 | | 99 |
| Aerobic cobaltochelatase, CobS | 319 | 99 | | 99 |
| Hypothetical protein,S088 | 255 | 98 | | 97 |
| Cobalamine biosynthesis protein, S068 | 317 | 99 | | 99 |
| Hypothetical protein,S069 | 146 | 99 | | 99 |
| Plasmid associated protein, S070 | 551 | 97 | | 98 |
| DNA repair protein, RadC | 165 | 99 | | 99 |
| Hypothetical protein, S092 | 113 | 96 | | 98 |
| Putative primase, S072 | 355 | 96 | | 96 |
| Hypothetical protein, S073 | 235 | 93 | | 94 |
| Hypothetical transposase | 105 |  | |  |
| ATP-dependent DNA helicase, RecQ | 709 |  | |  |
| DNA recombination-mediator protein A, DprA | 488 |  | |  |
| Hypothetical protein | 44 |  | |  |
| TraF | 314 | 94 | | 95 |
| TraH | 462 | 99 | | 99 |
| TraG | 1189 | 98 | | 98 |
| Exclusion system protein, Eex | 151 | 73 | | 71 |
| **DDE superfamily endonuclease^3^** | **136** |  | |  |
| Transcriptional activator, SetC | 172 | 99 | | 99 |
| Transcriptional activator, SetD | 100 | 99 | | 99 |
| LysM/invasin protein | 182 | 98 | | 98 |
| Hypothetical protein, S083 | 220 | 98 | | 98 |
| Hypothetical protein, S084 | 289 | 97 | | 97 |
| Hypothetical protein, SetQ | 83 | 100 | | 100 |
| cI prophage repressor protein, setR | 215 | 98 | | 98 |

^1^Contents of five hotspots and variable region Ⅰare shown in red

^2^Amino acid sequences of ORFs were compared for identity analysis

^3^Atypical insertions of genes in the backbone of the ICE are shown in boldface
