# Supplementary material for: Comparative genomic analysis of six new-found integrative conjugative elements (ICEs) in Vibrio alginolyticus
Source: BMC Microbiol. 2016 May 4;16:79. doi: 10.1186/s12866-016-0692-9 (PMC4857294; doi:10.1186/s12866-016-0692-9)
Supplement: Additional file 5: Table S5. — ORFs in ICEValHN437 and their similarity with related ICEs (DOCX 19 kb) [file 12866_2016_692_MOESM5_ESM.docx]

**Additional file 5: Table S5.** ORFs in ICE*Val*HN437 and their similarity with related ICEs

| Functions of genes^1^ | Length | % Identity^2^ | |
| --- | --- | --- | --- |
|  |  | SXT | ICE*Vch*Ban9 |
| Integrase, Int | 410 |  |  |
| Recombination directionality factor, Xis | 63 |  |  |
| Hypothetical protein, S002 | 90 | 96 | 95 |
| Rod shape determination protein, S003 | 324 | 99 | 99 |
| Hypothetical protein | 40 | 93 | 90 |
| Hypothetical protein, MobI | 147 | 99 | 98 |
| Error-prone repair protein, RumB | 422 | 99 | 92 |
| Error-prone repair protein, RumA | 149 | 98 | 98 |
| DNA polymerase III, S024 | 301 | 97 | 98 |
| **Transposase**^3^ | **459** |  |  |
| **Hypothetical protein**^3^ | **44** |  | **85** |
| Hypothetical protein, S025 | 62 | 63 | 63 |
| Hypothetical protein, S026 | 320 | 63 | 63 |
| DNA-methyltransferase subunit M, HsdM | 281 |  |  |
| Transposase | 318 |  |  |
| DNA-methyltransferase subunit M, HsdM | 571 |  |  |
| DNA specificity subunit S, HsdS | 423 |  |  |
| DNA restriction subunit R, HsdR | 1079 |  |  |
| Mrr restriction system protein | 284 |  |  |
| TraI | 716 | 93 | 95 |
| TraD | 606 | 99 | 99 |
| Conjugative transfer protein | 186 | 92 | 95 |
| TraJ | 211 | 99 | 99 |
| Hypothetical protein | 39 |  |  |
| Fic family protein | 368 |  |  |
| Hypothetical protein | 44 |  |  |
| HigA protein | 96 |  |  |
| TraL | 93 | 99 | 99 |
| TraE | 208 | 99 | 99 |
| TraK | 298 | 98 | 98 |
| TraB | 429 | 98 | 98 |
| TraV | 216 | 99 | 99 |
| TraA | 128 | 99 | 99 |
| Acetyltransferase | 168 |  |  |
| Conserved hypothetical protein | 88 |  |  |
| DsbC | 230 | 99 | 99 |
| TraC | 800 | 99 | 99 |
| Conjugative transfer protein | 115 | 99 | 98 |
| TrhF | 170 | 97 | 95 |
| TraW | 374 | 94 | 95 |
| TraU | 250 | 99 | 99 |
| TraN | 1230 | 98 | 98 |
| Hypothetical protein | 110 |  | 100 |
| Hypothetical protein | 220 |  | 100 |
| Hypothetical protein, S063 | 200 | 99 | 99 |
| Hypothetical protein, S089 | 108 | 97 | 99 |
| Single-stranded DNA-binding protein, Ssb | 139 | 96 | 99 |
| Recombination protein, Bet | 272 | 99 | 99 |
| Hypothetical protein, OrfZ | 47 | 96 | 91 |
| Recombination related exonuclease, Exo | 338 | 99 | 99 |
| Aerobic cobaltochelatase CobS subunit | 319 | 99 | 99 |
| Hypothetical protein, S088 | 255 | 97 | 98 |
| Cobalamine biosynthesis protein, S068 | 317 | 99 | 100 |
| Hpothetical protein, S069 | 146 | 96 | 96 |
| Plasmid associated protein, S070 | 551 | 97 | 96 |
| DNA repair protein, RadC | 165 | 99 | 99 |
| Hypothetical protein, S092 | 113 | 97 | 90 |
| Putative primase, S072 | 357 | 97 | 97 |
| Hypothetical protein, S073 | 43 | 95 | 97 |
| Transposase | 318 |  |  |
| Organic hydroperoxide resistance protein, OsmC | 123 |  |  |
| Transposase | 306 |  |  |
| Hypothetical protein | 65 |  |  |
| DDE endonuclease | 347 |  |  |
| Hypothetical protein | 461 |  |  |
| UDP-glucose dehydrogenase | 388 |  |  |
| Hypothetical protein | 210 |  |  |
| UTP--glucose-1-phosphate uridylyltransferas | 297 |  |  |
| Hypothetical protein | 113 |  |  |
| Phage lysine protein | 152 |  |  |
| Hypothetical protein | 100 |  |  |
| Transcriptional regulator | 189 |  |  |
| Hypothetical protein | 106 |  |  |
| Hypothetical protein | 135 |  |  |
| Hypothetical protein | 348 |  |  |
| L-Rha alpha-1,3-L- rhamnosyltransferase | 672 |  |  |
| Hypothetical protein | 326 |  |  |
| Glycosyl transferase | 581 |  |  |
| Capsular polysaccharide ABC transporter, KpsT | 216 |  |  |
| Sulfate permease | 578 |  |  |
| Sulfate adenylyltransferase subunit 2 | 304 |  |  |
| Adenylylsulfate kinase | 209 |  |  |
| Acetyltransferase | 320 |  |  |
| Sulfate adenylyltransferase subunit 1 | 468 |  |  |
| RecD-like DNA helicase | 54 |  |  |
| Hypothetical protein | 88 |  |  |
| Transposase | 468 |  |  |
| Transposase | 251 |  |  |
| Hypothetical protein | 232 |  |  |
| TraF | 314 | 96 | 96 |
| TraH | 462 | 99 | 99 |
| TraG | 1189 | 99 | 99 |
| Exclusion system protein, Eex | 143 | 71 | 99 |
| Transcriptional activator, SetC | 177 | 99 | 99 |
| Transcriptional activator, SetD | 99 | 100 | 99 |
| LysM/invasin protein | 182 | 99 | 99 |
| Hypothetical protein, S083 | 220 | 99 | 99 |
| Hypothetical protein, S084 | 289 | 98 | 99 |
| Hypothetical protein, SetQ | 83 | 100 | 100 |
| cI prophage repressor protein, SetR | 215 | 97 | 102 |

^1^Contents of five hotspots are shown in red

^2^Amino acid sequences of ORFs were compared for identity analysis

^3^Atypical insertions of genes in the backbone of the ICE are shown in boldface
