# Supplementary figures and images for: Comparative genomic analysis of six new-found integrative conjugative elements (ICEs) in Vibrio alginolyticus
Source: BMC Microbiol. 2016 May 4;16:79. doi: 10.1186/s12866-016-0692-9 (PMC4857294; doi:10.1186/s12866-016-0692-9)

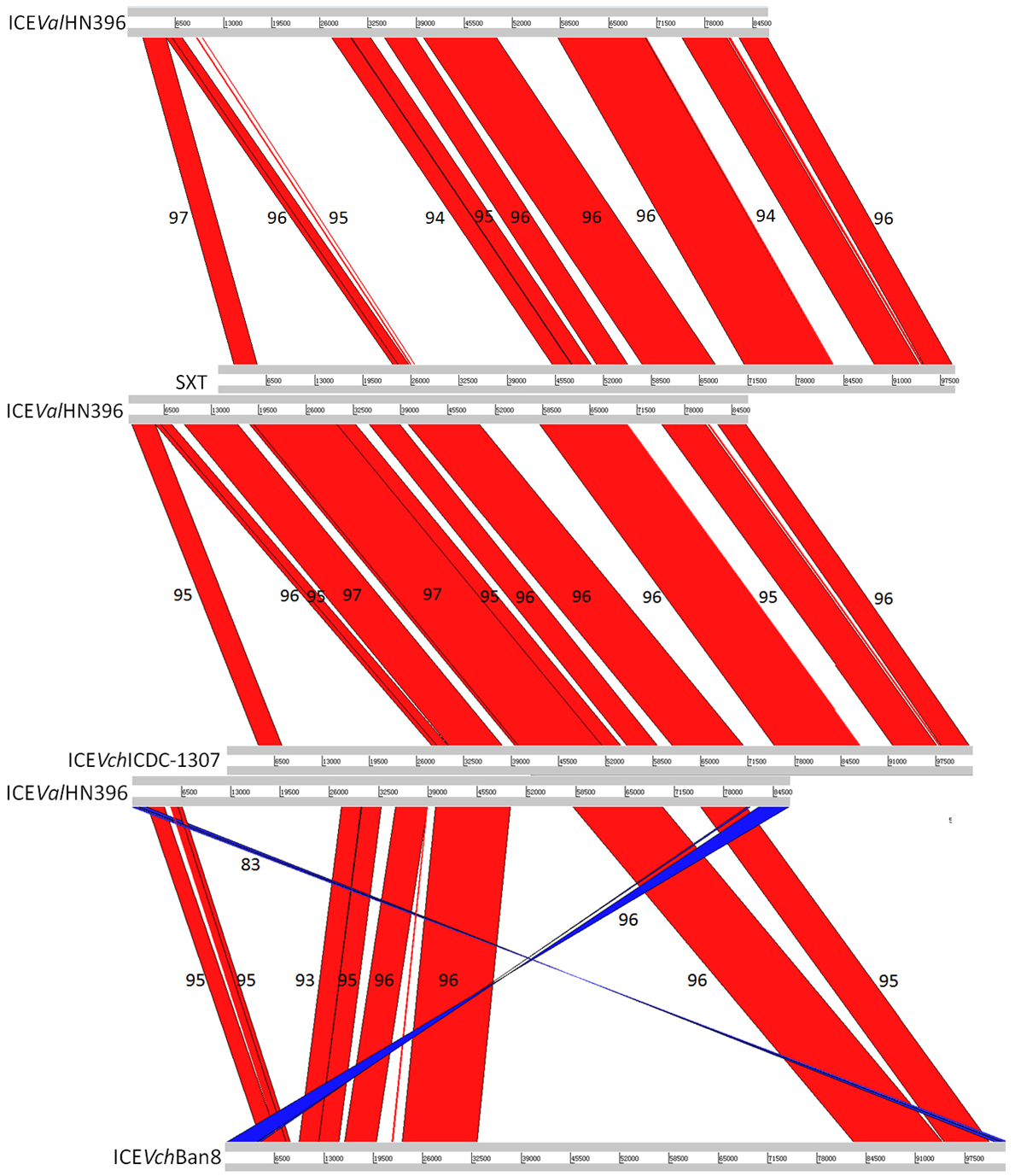

Supplement: Additional file 6: Figure S1. — Alignment generated using WebACT of ICEValHN396, SXT, ICEVchICDC-1307 and ICEVchBan8. Comparisons between the regions < 80 bp are filtered. Numbers show the identity values of the compared regions. Red areas indicate homologous regions; blue areas indicate inversions. (TIF 522 kb) [file 12866_2016_692_MOESM6_ESM.tif]

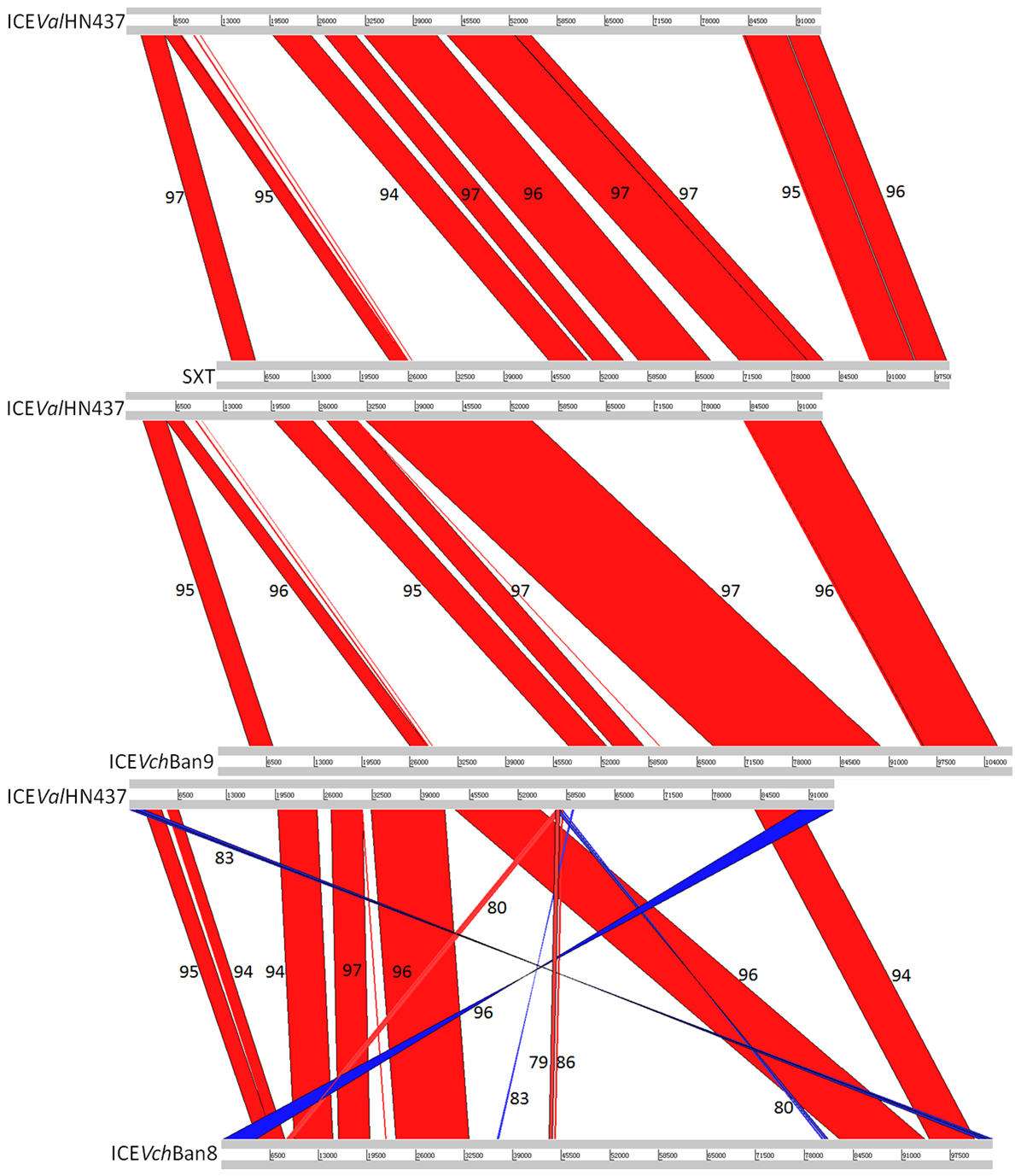

Supplement: Additional file 7: Figure S2. — Alignment generated using WebACT of ICEValHN437, SXT, ICEVchBan9 and ICEVchBan8. Comparisons between the regions < 80 bp are filtered. Numbers show the identity values of the compared regions. Red areas indicate homologous regions; blue areas indicate inversions. (TIF 493 kb) [file 12866_2016_692_MOESM7_ESM.tif]
