# Supplementary material for: Comparative genomic analysis of six new-found integrative conjugative elements (ICEs) in Vibrio alginolyticus
Source: BMC Microbiol. 2016 May 4;16:79. doi: 10.1186/s12866-016-0692-9 (PMC4857294; doi:10.1186/s12866-016-0692-9)
Supplement: Additional file 8: Table S6. — Primers used for gap filling in this study. (DOCX 15 kb) [file 12866_2016_692_MOESM8_ESM.docx]

**Additional file 8: Table S6.** Primers used for gap filling in this study

| Primer name | Nucleotide sequence (5′ to 3′)*^a^* | Gap filling for |
| --- | --- | --- |
| A056-Sca21F | TTGCGTCATTACGTCCAGCAAG | ICEValA056-1 |
| A056-Sca41-34R | GTAGAGCAGCCATTCGTAACGT |  |
| A056-Sca41-34F | GAGCTGTGCGGCAGCGCTCAGT | CEValA056-1 |
| A056-Sca19R | AGAGCAGCCATTCGTAACGTTG |  |
| A056-Sca19F | CTGGAGGTGATGCAGCAGTTGA | ICEValA056-1 |
| A056-Sca26R | GTCTGAAACGGACTTCGATCGG |  |
| A056-Sca26F | CCGAGGTCAATGTAACGTGCTT | ICEValA056-1 |
| A056-Sca38R | TGGAAGGCATTGATGAAGACAAC |  |
| A056-Sca38F | ATGGCAGACGCACACTAAGC | ICEValA056-1 |
| A056-Sca2R | AATGCGCCACTGGTGCCAT |  |
| A056-Sca10F | TGAAGTGCCTTTATCTGCCAT | ICEValA056-2 |
| A056-Sca24R | CCTCTGGATACACATCTTATTGTC |  |
| A056-Sca24F | ATGGCTACCTAATGGAACTACC | ICEValA056-2 |
| A056-Sca25R | GGATGGAATGCATTGATGAAGAC |  |
| A056-Sca25F | ATGGCAGACGCACACTAAGC | ICEValA056-2 |
| A056-Sca3R | GAGTACGACCGTTGTACTAACG |  |
| E0601-Sca50F | GGCTGATCTTCTGAACAAATC | ICE*Val*E0601 |
| E0601-Sca44R | ACAGTGCCTATAGAGATCCG |  |
| E0601-Sca44F | ACTTACTCGTGGCTGCAACCCT | ICE*Val*E0601 |
| E0601-Sca5R | CTCGCTTGAGCCGTATGGCATC |  |
| HN396-Sca7F | GTTGTCGTTCAGCCACTTCAG | ICE*Val*HN396 |
| HN396-Sca51R | GCCCTGTGTGCGAGATTATGG |  |
| HN437-Sca16F | GACAGCATTACCTATTATGCTCA | ICE*Val*HN437 |
| HN437-Sca40R | CCGCATCCACATATAGGGCA |  |
| HN437-Sca40F | ACACTGTATGTAGATCCAGCT | ICE*Val*HN437 |
| HN437-Sca39R | AGTTCGAGTAGCTAGCCGTTC |  |
| HN492-Sca44F | GACTTACTCGTGGCTGCAAC | ICE*Val*HN492 |
| HN492-Sca39R | CGAACTTCAGGCTTACCTAGC |  |
| HN492-Sca39F | AAGCATCTTCAATGATCACGG | ICE*Val*HN492 |
| HN492-Sca26R | TCAACCGATGACGATATCACTG |  |
